# Synaptic Plasticity After Focal Cerebral Ischemia Was Attenuated by Gap26 but Enhanced by GAP-134

**DOI:** 10.3389/fneur.2020.00888

**Published:** 2020-08-26

**Authors:** Kailing Yang, Ying Zhou, Lequan Zhou, Fuman Yan, Li Guan, Haimei Liu, Wei Liu

**Affiliations:** ^1^Department of Physiology, School of Basic Medical Science, Guangzhou University of Chinese Medicine, Guangzhou, China; ^2^The Research Center of Basic Integrative Medicine, Guangzhou University of Chinese Medicine, Guangzhou, China

**Keywords:** focal ischemia, Gap26, GAP-134, synaptic plasticity, Cx43, SYN, GAP-43

## Abstract

**Objective:** Synaptic plasticity is critical for neurorehabilitation after focal cerebral ischemia. Connexin 43 (Cx43), the main component of the gap junction, has been shown to be pivotal for synaptic plasticity. The objective of this study was to investigate the role of the Cx43 inhibitor (Gap26) and gap junction modifier (GAP-134) in neurorehabilitation and to study their contribution to synaptic plasticity after focal ischemia.

**Methods:** Time course expression of both total and phosphorylated Cx43 (p-Cx43) were detected by western blotting at 3, 7, and 14 d after focal ischemia. Gap26 and GAP-134 were administered starting from 3 d post focal ischemia. Neurological performances were evaluated by balance beam walking test and Y-maze test at 1, 3, and 7 d. Golgi staining and transmission electron microscope (TEM) detection were conducted at 7 d for observing dendritic spine numbers and synaptic ultrastructure, respectively. Immunofluorescent staining was used at 7 d for detection of synaptic plasticity markers, including synaptophysin (SYN) and growth-associated protein-43 (GAP-43).

**Results:** Expression levels of both total Cx43 and p-Cx43 were increased after focal cerebral ischemia, peaking at 7 d. Compared with the MCAO group, Gap26 worsened the neurological behavior and decreased the dendritic spine number while GAP-134 improved the neurobehavior and increased the number of dendritic spines. Moreover, Gap26 further destroyed the synaptic structure, concomitant with downregulated SYN and GAP-43, whereas GAP-134 alleviated synaptic destruction and upregulated SYN and GAP-43.

**Conclusion:** These findings suggested that Cx43 or the gap junction was involved in synaptic plasticity, thereby promoting neural recovery after ischemic stroke. Treatments enhancing gap junctions may be potential promising therapeutic measures for neurorehabilitation after ischemic stroke.

## Introduction

Ischemic stroke is a worldwide leading cause for morbidity, mortality, and disability. Until now, tissue plasminogen activator (tPA), which should be treated within 4.5 h, is the most effective strategy for acute ischemia (1). However, a lot of patients do not have the opportunity to be treated within 4.5 h (acute therapeutic window). As a result, to improve life quality and lighten corresponding burdens, treatments for promoting neural function during the subacute phase is worth exploring more. The key molecular basis of neural function after cerebral ischemia is synaptic plasticity (2).

Synaptic plasticity refers to a reparative process and contributes to improvement of neurological behavior. Numerous treatments which promoted synaptic plasticity have been demonstrated to be protective against ischemic injury. For example, exercise training has been reported to enhance synaptic plasticity and thereby promote rehabilitation after ischemic stroke (3). In addition, electroacupuncture and Buyang Huanwu decoction (a classical formula in traditional Chinese medicine) could positively alter synaptic plasticity and are beneficial for restoration after ischemic stroke (4, 5). If the potential molecular mechanisms of synaptic plasticity can be elucidated, it will trigger the appearance of many effective measures for treating ischemic stroke patients to improve their neurological behavior. Therefore, there is a great need to investigate the mechanisms underlying synaptic plasticity after cerebral ischemia.

Astrocytes are increasingly recognized to be involved in neurorehabilitation after ischemia (6). It has been shown that astrocytes affected the reconstruction of synaptic connections and strongly promoted synaptic connections of neurons (7). Gap junctions among astrocytes have been demonstrated to facilitate cellular communication, such as electrical current flow and exchange of cytoplasmic molecules, energy substrates, and also some trophic factors, which would probably promote neurorehabilitation during the recovery period after focal ischemia. The most ubiquitously expressed hemichannel, connexin 43 (Cx43), has been widely accepted as an important target for the treatment of ischemic stroke (8). However, the potential role of Cx43 in synaptic plasticity after cerebral ischemia has yet to be determined.

Therefore, we firstly investigated the expression levels of Cx43 after focal ischemia, then explored the effects of the Cx43 inhibitor (Gap26) and gap junction modifier (GAP-134) on neurorehabilitation (neurological function, dendritic spine morphology, and numbers), and synaptic plasticity (synaptic structure and related proteins).

## Materials and Methods

### Animals

All experiments were approved by the Animal Care and Use Committee of the Guangzhou University of Chinese Medicine. All studies were conducted in accordance with the National Institutes of Health Guidelines for the Care and Use of Laboratory Animals. Adult male Sprague-Dawley rats (250–300 g) were obtained from the Experimental Animal Center of the Guangzhou University of Chinese Medicine (Guangzhou, China).

### Middle Cerebral Artery Occlusion (MCAO) Model

Rats were anesthetized with 10% chloral hydrate (350 mg/kg, intraperitoneally). Subsequently, a mid-line incision was made in the neck of rats. The right common carotid artery (CCA), external carotid artery (ECA), and internal carotid artery (ICA) were isolated. The ECA was cut approximately 4 mm above the common carotid artery bifurcation. A 4.0-s nylon monofilament suture was inserted into ICA through the stump until resistance. In this way, the blood flow to the middle cerebral artery (MCA) was blocked. 2 h later, the filament was taken out for reperfusion, and the incision was ligated. The rats of the sham group underwent the same surgery process but without blood flow blockade. After being awake, rats were evaluated for the neurological deficits according to the modified Longa scoring system: 0, no neurological deficit; 1, failure to fully extend left forepaw; 2, circling to the left; 3, falling to the left; 4, loss of spontaneous walking with a depressed level of consciousness; and 5, dead. The rats scored 1, 2, and 3 were successful models and randomized into three groups: MCAO, Gap26, and GAP-134.

### Drug Administration

Gap26 (Tocris, UK) was dissolved in saline and given intraperitoneally at the dose of 25 μg/kg once a day (9). GAP-134 (MCE, USA) was dissolved in distilled water and given intraperitoneally at the dose of 3 mg/kg twice a day (10). To avoid the influence in the acute phase, we chose to apply Gap26 or GAP-143 from day 3 after ischemia, when the infarction volume stabilized and the repair period began (11). The sham and MCAO groups were treated with distilled water.

### Balance Beam Walking Test

The coordination and integration of motor movement were evaluated using the balance beam walking test at 1, 3, and 7 d after surgery. The beam was 3 cm in width, 90 cm in length, and 50 cm in height above the ground. The rats were trained to walk on the beam from one end to the other before surgery to ensure that all rats can go through successfully. A six-point scoring system was developed by Ohlsson as the following: 0, the rat was able to balance and walk on the beam with steady posture; 1, the rat traversed the beam with <50% foot slips; 2, the rat traversed the beam with more than or equal to 50% foot slips; 3, the rat crossed the beam, but the affected limbs cannot aid in forward locomotion; 4, the rat fell down while walking; 5, the rat was unable to cross the beam but remained sitting on the beam; and 6, the rat fell down immediately (12). A higher score indicated a more severe injury. The test was conducted three times for each rat, and averaged scores were calculated for statistical analysis.

### Y-Maze Test

Spontaneous alternation performance, an index of spatial working memory, was tested by Y-maze at 1, 3, and 7 days after surgery. Each arm of the Y-maze was 80 cm long and 16 cm wide and converged at an equal angle of 120°. Each rat was placed at the center of the maze and allowed to move freely through the maze for 8 min. The series of arm entries, including total number and sequence of arm entries, were recorded. An alternation was defined as entries in all three arms on consecutive occasions. The percentage of alternation was calculated as the following: (number of alternations/number of total arm entries−2) × 100% (13).

### Golgi-Cox Staining

At 7 d after MCAO, rats were sacrificed, and brains were removed as soon as possible. Rapid Golgi Stain Kit (FD, USA) was applied for tissue preparation and staining procedures. The whole Golgi-Cox staining procedures were conducted in strict accordance with instructions. Using a freezing microtome (Leica, Germany), each block was then cut coronally into 100-μm-thick serial sections starting at +1.00 to 5.00 mm from the bregma. The sections were mounted onto slides and observed under a laser scanning confocal microscope (ZEISS, Germany). The dendritic spine density of neurons in hippocampal CA1, CA3, and DG were analyzed, respectively, by Image-Pro Plus 6.0.

### Transmission Electron Microscopy (TEM) Detection

At 7 d after MCAO, rats were deeply anesthetized and perfused with 0.9% saline and 4% paraformaldehyde successively. Brains were taken out, and the hippocampus was separated and cut into 1-cubic-millimeter fragments and fixed in 2.5% glutaraldehyde for 12 h followed by 1% osmic acid for 4 h. Specimens were dehydrated with acetone and sectioned into 70-nm slices. Finally, the slices were stained with 2% uranyl acetate and lead citrate. Each slice was captured under the TEM (Hitachi, Japan).

### Immunofluorescent Staining

Rats were deeply anesthetized and perfused with 0.9% saline followed by 4% paraformaldehyde at 7 d after MCAO. Brains were taken out and immersed in sucrose for dehydration (sucrose of 15 and 30% successively). Coronal sections were cut at 10-μm thickness by freezing microtome (Leica, Germany). After being permeabilized with 0.3% Triton X-100 and blocked by goat serum working liquid, sections were incubated at 4°C overnight with primary antibodies: synaptophysin (SYN, 1:200, Millipore, USA) and growth-associated protein-43 (GAP-43, 1:250, Millipore, USA). The next day after being washed in Tris-buffered saline (TBS), sections were incubated with goat anti-mouse/rabbit IgG H&L secondary antibody (1:200, Abcam, USA) for 2 h at room temperature and mounted using anti-fade solution with DAPI (Solarbio, Shanghai, China). The stained sections were captured under a fluorescent laser scanning confocal microscope (ZEISS, Germany) and analyzed with Image-Pro Plus 6.0.

### Western Blotting

After rats were deeply anesthetized and transcardially perfused with 0.9% cold saline, brains were quickly removed, and the hippocampi were collected and frozen separately in liquid nitrogen. Samples were homogenized in RIPA buffer (Sigma, USA) containing a protease inhibitor and phosphatase inhibitor and stood on ice for 1 h, then centrifuged at 12,000 rpm for 20 min at 4°C. The supernatant was collected, and the protein concentration was quantified using BCA Protein Assay (Thermo, USA). Proteins (30 μg) were loaded onto 4% stacking 12% separating SDS-PAGE gel for electrophoresis and transferred onto polyvinylidene fluoride membranes (Millipore, USA). After being blocked with 5% BSA (Roche, Germany), membranes were incubated overnight at 4°C with primary antibodies (anti-Cx43, 1:1000, Abcam, USA; anti-p-Cx43, 1:1000, Abcam, USA; anti-β-actin, 1:5000, Abcam, USA). After incubation for 1 h at room temperature with secondary antibodies (anti-rabbit, 1:5000, Abcam, USA; anti-mouse, 1:5000, Abcam, USA), the membranes were developed with ECL solution (Millipore, USA) and exposed to an X-ray film. For quantification, the intensity of bands was measured using the ImageJ software (National Institutes of Health, USA).

### Statistical Analysis

All data were expressed as mean ± SEM. Statistical analysis was performed by SPSS 17.0. The multiple comparisons among groups were made using one-way analysis of variance (ANOVA). The pairwise comparison of mean values was tested by least significant difference (LSD). Significance level was set at *P* < 0.05.

## Results

### Time Course Expression of Cx43

**Figure 1:**
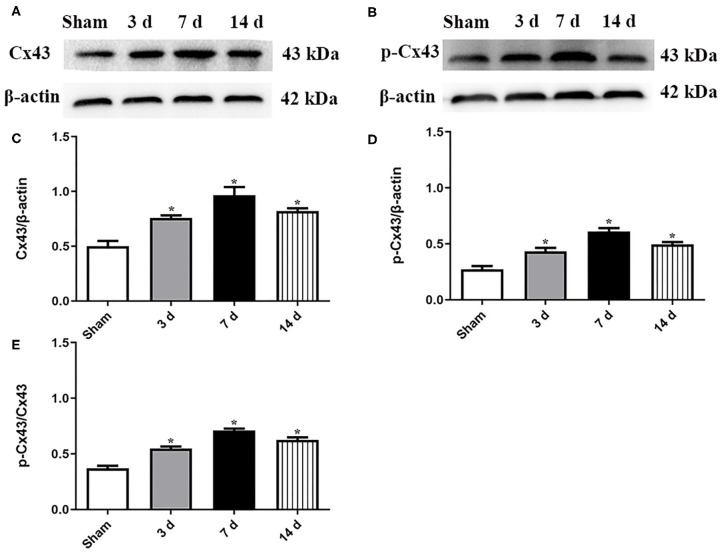
Expression levels of both Cx43 and p-Cx43 were detected at 3, 7, and 14 d after MCAO. Data were presented as mean ± SEM, *n* = 6. Representative Western blot bands and schematic diagram showed that total Cx43 **(A,C)**, p-Cx43 **(B,D**), and ratio of p-Cx43/Cx43 **(E)** were increased after MCAO and peaked at 7 d (*P < 0.05 vs. sham).

As shown in Figure 1, the expression levels of both total Cx43 and p-Cx43 in the hippocampi were significantly upregulated at 3, 7, and 14 d after MCAO. In addition, the ratio of p-Cx43/Cx43 showed a similar tendency at 3, 7, and 14 d after MCAO (*P* < 0.05, Figures 1A–E), suggesting that Cx43 might be involved in recovery after MCAO. Notably, the upregulation peaked at 7 d, so subsequent experiments were conducted at 7 d after MCAO.

### Effects of Gap26 and GAP-134 on Neurological Behavior

**Figure 2:**
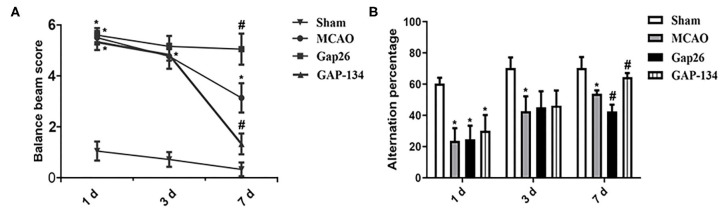
Effects of Gap26 and GAP-134 on balance beam walking test and Y-maze test at 1, 3, and 7 d after MCAO. Data were presented as mean ± SEM, *n* = 6. **(A)** Compared with MCAO, Gap26 significantly increased the balance beam score while GAP-134 decreased it at 7 d. **(B)** Compared with MCAO, Gap26 downregulated the alternation percentage; however, GAP-134 upregulated it at 7 d (*P < 0.05 vs. sham, ^#^*P* < 0.05 vs. MCAO).

Neurological behavior detected by balance beam walking test and Y-maze test was analyzed at 1, 3, and 7 d, respectively (Figure 2). For the balance beam walking test, scores for the MCAO group, Gap26 group, and GAP-134 group were all dramatically higher when compared with the sham group at 1 d (*P* < 0.05, Figure 2A). Due to drug treatments just beginning from 3 d, no wonder no difference existed between the MCAO group and the Gap26/GAP-134 group at 3 d after focal ischemia (*P* > 0.05, Figure 2A). However, at 7 d the score for the Gap26 group was significantly higher than that for the MCAO group (*P* < 0.05, Figure 2A), while the GAP-134 group exhibited a markedly lower score compared with the MCAO group (*P* < 0.05, Figure 2A).

As to the Y-maze test, rats in the MCAO group, Gap26 group, and GAP-134 group all demonstrated an obviously decreased alteration percentage compared to the sham group at 1 d (*P* < 0.05, Figure 2B). There was no significant difference at 3 d between the MCAO group and Gap26/GAP-134 group (*P* > 0.05, Figure 2B), as drug treatments just began from 3 d. However, at 7 d the alteration percentage of the Gap26 group was significantly lower than that of the MCAO group (*P* < 0.05, Figure 2B), while GAP-134 treatment markedly increased the alteration percentage compared with the MCAO group (*P* < 0.05, Figure 2B).

### Effects of Gap26 and GAP-134 on Hippocampal Dendritic Spines

**Figure 3:**
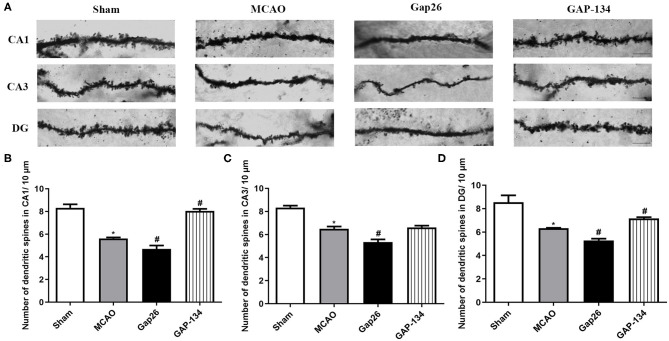
Effects of Gap26 and GAP-134 on dendritic morphology detected by Golgi-Cox staining at 7 d after MCAO. Data were presented as mean ± SEM, *n* = 3. **(A)** Representative Golgi-Cox-stained sections in hippocampal CA1, CA3, and DG. Scale bar = 20 μm. **(B–D)** Quantitative analysis of spine density in CA1, CA3, and DG. The spine number was decreased in the MCAO, and Gap26 treatment further decreased it in CA1, CA3, and DG while GAP-134 alleviated the decrease in CA1 and DG (*P < 0.05 vs. sham, ^#^*P* < 0.05 vs. MCAO).

Dendritic spine morphology and number in hippocampus were examined and quantified by Golgi-Cox staining at 7 d after focal ischemia. As shown in Figure 3A, in the sham group the dendritic spines were regular and intense; however, fewer spines were observed in the MCAO group (*P* < 0.05, Figures 3B–D). Administration with Gap26 further decreased the spine density in CA1, CA3, and DG (*P* < 0.05, Figures 3B–D); however, the GAP-134 group significantly increased the dendritic spine density in CA1 and DG (*P* < 0.05, Figures 3B,D).

### Effects of Gap26 and GAP-134 on Synaptic Ultrastructure in the Hippocampus

**Figure 4:**
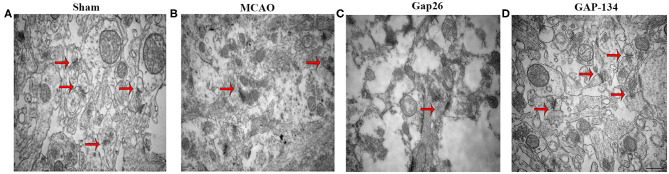
Effects of Gap26 and GAP-134 on ultrastructural changes in the hippocampus at 7 d after MCAO. Representative synapse ultrastructure of all groups. The structure of synapses was intact and clear in the sham group **(A)**; however, destructed synapses were seen after MCAO **(B)**, demonstrated by dim cleft, fusion of synaptic space, and etc. Gap26 further deteriorated it **(C)** while GAP-134 improved it **(D)**. Synapses were indicated in red arrows. Scale bar = 500 nm.

The ultrastructure of hippocampal synapses was analyzed using TEM at 7 d after focal ischemia. As shown in Figure 4A, the synapses in the sham group remained intact and clear. The structure was destroyed by MCAO (Figure 4B), demonstrated by dim synaptic cleft, fusion of synaptic space, loss of synaptic vesicles, incomplete synaptic structure, and swelling of the presynaptic terminal. Administration with Gap26 further deteriorated the destruction of synapses (Figure 4C). However, the destruction induced by MCAO was obviously improved in the GAP-134 group (Figure 4D).

### Effects of Gap26 and GAP-134 on Expressions of SYN and GAP-43

Immunofluorescent staining was applied to detect and quantify the expressions of SYN and GAP-43 in CA1, CA3, and DG area of the hippocampus at 7 d after focal ischemia.

**Figure 5:**
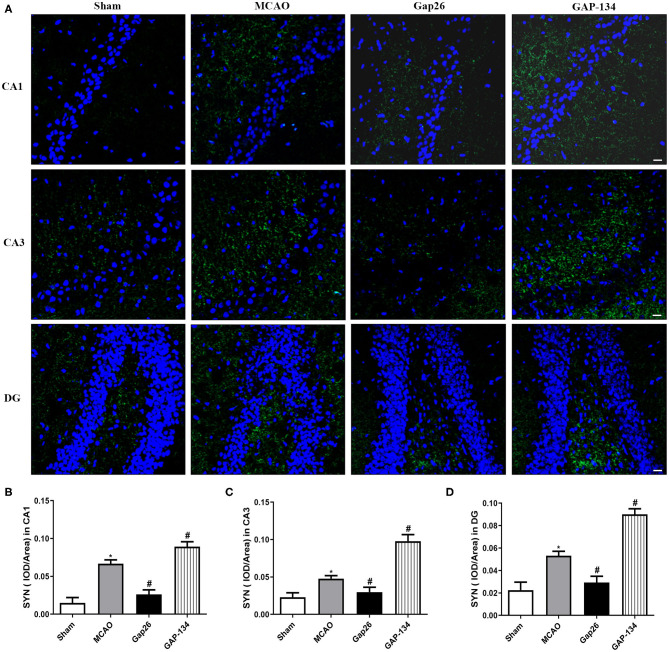
Effects of Gap26 and GAP-134 on the expression of SYN at 7 d after MCAO. Data were shown as mean ± SEM, *n* = 6. **(A)** Representative pictures of SYN in hippocampal CA1, CA3, and DG. Blue depicts staining of DAPI (cell nuclei), and green depicts staining of SYN. Scale bar = 20 μm. **(B–D)** Quantitative analysis showed that expression of SYN was decreased by Gap26 but increased by GAP-134 in hippocampal CA1, CA3, and DG (*P < 0.05 vs. sham, ^#^*P* < 0.05 vs. MCAO).

Representative pictures of SYN are shown in Figure 5A, and semi-quantifications were conducted in CA1, CA3, and DG, respectively (Figures 5B–D). As shown in Figure 5, in the sham group only weak expression of SYN was observed in hippocampal CA1, CA3, and DG. The expression of SYN was dramatically increased in the MCAO group (*P* < 0.05, Figure 5). When compared with the MCAO group, administration with Gap26 significantly decreased its expression (*P* < 0.05, Figure 5), while GAP-134 markedly upregulated the expression of SYN (*P* < 0.05, Figure 5).

**Figure 6:**
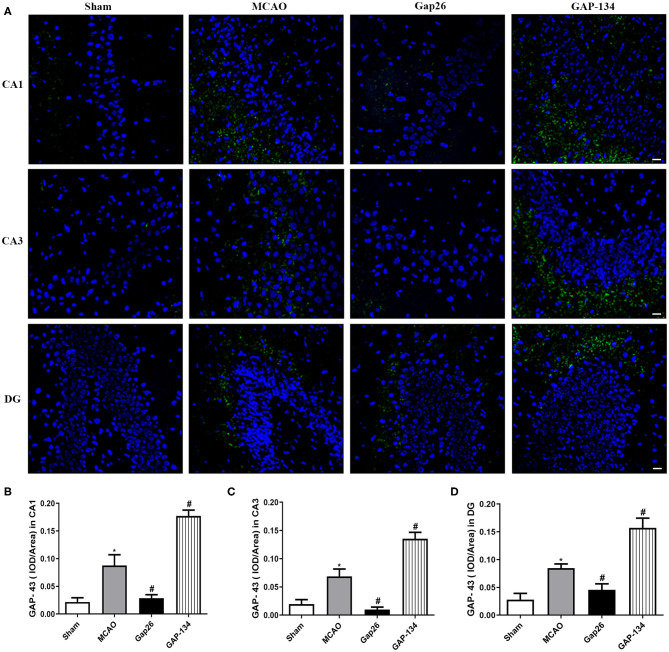
Effects of Gap 26 and GAP-134 on the expression of GAP-43 at 7 d after MCAO. Data were shown as mean ± SEM, *n* = 6. **(A)** Representative pictures of GAP-43 in hippocampal CA1, CA3, and DG. Blue depicts staining of DAPI (cell nuclei), and green depicts staining of SYN. Scale bar = 20 μm. **(B–D)** Quantitative analysis showed that the expression of GAP-43 was downregulated by Gap26 but upregulated by GAP-134 in hippocampal CA1, CA3, and DG (*P < 0.05 vs. sham, ^#^*P* < 0.05 vs. MCAO).

Similarly, in CA1, CA3, and DG, the area of hippocampus GAP-43 staining was significantly increased in the MCAO group (*P* < 0.05, Figure 6), while Gap26 obviously decreased the GAP-43 expression compared with the MCAO group (*P* < 0.05, Figure 6). However, GAP-134 dramatically increased the expression of GAP-43 (*P* < 0.05, Figure 6).

## Discussion

In the present study, we demonstrated that expression levels of both total and phosphorylated Cx43 were increased after focal ischemia. Inhibition of Cx43 with Gap26 significantly worsened the neurobehavioral behaviors, decreased the dendritic spine density, deteriorated the destroyed synaptic structure, and downregulated the expression levels of SYN and GAP-43. By contrast, GAP-134 alleviated neurological deficits, increased the dendritic spine density, and protected the synaptic morphology, which were accompanied by increases in SYN and GAP-43. These findings indicated that Cx43 or the gap junction was involved in synaptic plasticity after focal ischemia.

Cx43, a major component of astrocytic gap-junction channels, is abundantly expressed in astrocytes (8). The role of Cx43 has been intensively investigated in acute ischemic stroke (9, 14). Ischemic injury activated astrocytic hemichannels, which caused an uncontrolled release of ATP and glutamate and an overload of Ca^2+^, leading to tissue excitotoxicity, inflammation, and ultimately irreversible brain damage (15). Gap26 treatment led to a reduction in both Cx43 expression and activity, therefore reducing the infarct volume and improving the neurological function (9). Gap junction blocker carbenoxolone or octanol played protective roles in ischemic preconditioning or cerebral ischemia (16, 17). Recent studies showed that inhibition of Cx43 hemichannels alleviated cerebral ischemia via the toll-like receptor (TLR4) or JAK2/STAT3 pathway (18, 19). Notably, the intervention of Cx43 or the gap junction occurred immediately after ischemia (during acute phase) in numerous experiments (9, 16–19). In contrast, few studies investigated their roles in the subacute phase after focal ischemia. Here we aimed at elucidating the roles of Gap26 and GAP-134 in the repair period (subacute phase) after focal cerebral ischemia.

In the present study, we evaluated the temporal expression of Cx43 (both total and phosphorylated form) during the subacute phase after MCAO and found that they were increased with a time-dependent manner and peaked at 7 d post focal ischemia. Similarly, previous studies have reported that the Cx43 and p-Cx43 immunoreactivity was significantly amplified in the subacute model (20). We speculated that an increase in Cx43 expression might be an effort to strengthen the function of gap junctional intercellular communication, which would generate protective function and improve neural recovery after focal ischemia. This speculation was comparable with recent studies in which Cx43 mediated the function of erythropoietin (EPO), improving neurological functions and reducing astrocytic activation (21).

Our data showed that administration with Gap26 during the subacute phase after cerebral ischemia deteriorated neurological deficits and downregulated dendritic spine density, indicating that Cx43 played an essential role in neurorehabilitation. This seems to be paradoxical with previous studies where Gap26 treated immediately after ischemia exhibited protective effects (9). This may be explained due to different medication times (acute phase vs. subacute phase). During the acute phase, harmful molecules such as free radicals and excitatory amino acids propagated through gap junctions and enlarged ischemic injury. However, during the subacute phase the infarct volume has been stabilized and several nutrient factors were released and diffused via the gap junction so as to protect against ischemic injury. This explanation was supported by previous studies where Cx43 overexpression restored the neurite growth and promoted neuronal recovery (22). Moreover, cortical neurons cultured with astrocytes treated with OGD/R were rescued by the overexpression of Cx43 and prone to grow better, which attributed to the role of Cx43 in cell–cell communication (22). Our data showed that GAP-134 alleviated neurological deficits induced by MCAO and increased the number of dendritic spines, indicating that gap junctions were critical for brain repair after cerebral ischemia. Our study is the first to show the protective role of Cx43 or the gap junction during the subacute phase after cerebral ischemia, which suggests that enhancing the expression of Cx43 or function of the gap junction can be a promising candidate to promote neurorehabilitation after cerebral ischemia.

Synaptic plasticity is a critical process for neural repair after focal ischemia. Cx43 has been shown to be important in the neuron–glia interactions required for whisker-related sensory functions and plasticity (23). Moreover, it has been demonstrated that mice deficient for Cx43 had impaired long-term synaptic plasticity, indicating that Cx43 plays an important role in synaptic plasticity (24). By using TEM, we assessed the synaptic ultrastructure at 7 d post focal ischemia and found that the destroyed synaptic ultrastructure induced by MCAO was more severe after treatment with Gap26; however, the synaptic disorganization was significantly ameliorated by GAP-134.

SYN and GAP-43 are synaptic plasticity markers and play pivotal roles in neurorehabilitation. SYN is a calcium-bound protein located in the presynaptic vesicle membrane, which participates in the regulation of synaptic plasticity. GAP-43 is a protein involved in neurite outgrowth during axon regeneration (25). We quantitatively measured these two proteins to further determine the effects of Gap26 or GAP-134 on synaptic plasticity. The staining indicated that Gap26 decreased the expression of these two proteins while GAP-134 increased their expression. These data were comparable with previous studies that upregulation of SYN and GAP-43 was closely associated with the enhanced synaptic and protective effect of retinoic acid, electroacupuncture, and willed-movement training (26–28).

Notably, there are reports showing that synaptophysin was recognized as a marker for axonal damage under different neuropathological conditions (29), which seemed to be paradoxical with our study. This contradiction may be explained as the following: synaptophysin, which is a major small synaptic vesicle protein, is synthesized in the neuronal cell body and then delivered by anterograde transport of the axon to numerous synapses. If the axon is damaged, synaptophysin transportation would be blocked and piled up, thus being a marker for axonal damage. Consistent with our results, there are numerous investigations that SYN was involved in hippocampus-dependent cognition after ischemic stroke, and upregulated SYN expression promoted motor function recovery (30).

One limitation of this study was no specific intervention for Cx43 involved to clearly elucidate its role in neurorehabilitation during the subacute phase after cerebral ischemia. Genetic knockdown was unfeasible, as Cx43 was generally considered to be harmful in the acute phase after cerebral ischemia, which would make the role of Cx43 in the subacute phase obscure. Gap26 (Cx43 mimetic peptide), once regarded as a selective Cx43 blocker, has been used in numerous experimental models (31, 32). The mechanism of Gap26 was reported to be the inhibition of cell coupling occurring across gap junctions (33). Recently, some studies showed that Gap26 also inhibited the openings of gap junctions (18, 19). Besides, no direct Cx43 activator was available. However, the application of GAP-134 oppositely verified the effects of Gap26 to some extent, because inhibition of Cx43 and the gap junction by Gap26 was contrary to the function of GAP-134. GAP-134 was a recently developed specific activator of the glial gap junction and used as an anti-arrhythmic peptide (34). Notably, GAP-134 has been used in the central nervous system and could increase the frequency of spontaneous epileptiform activities (35). Our data indicated that GAP-134 significantly improved the balance beam walking test and Y-maze test, increased the dendritic spine densities, and alleviated the synaptic destruction, concomitant with upregulated SYN and GAP-43, suggesting that GAP-134 may enhance neural recovery via promoting synaptic plasticity and thus might be a potential treatment for ischemic injury. However, more studies are needed in future.

## Conclusions

Our present study showed that Cx43 or the gap junction acted as a novel regulator for neurorehabilitation. Ischemia induced Cx43 upregulation, and treatment with its inhibitor Gap26 worsened the neurological behavior, deteriorate the synapse ultrastructure, and decreased the expression of SYN and GAP-43, while GAP-134 showed opposite effects. Therefore, Cx43 or the gap junction may be an effective therapeutic target during the subacute treatment window.

## Data Availability Statement

All datasets generated for this study are included in the article/Supplementary Material.

## Ethics Statement

The animal study was reviewed and approved by Animal Care and Use Committee of the Guangzhou University of Chinese Medicine.

## Author Contributions

KY and YZ performed the experiments and data analysis. LZ, FY, LG, and HL contributed significantly to statistical analysis and manuscript preparation. WL designed the study, provided constructive opinions during the experiments, and wrote the manuscript. All authors contributed to the article and approved the submitted version.

## Conflict of Interest

The authors declare that the research was conducted in the absence of any commercial or financial relationships that could be construed as a potential conflict of interest.

## Abbreviations

Cx43: connexin 43
MCAO: middle cerebral artery occlusion
TEM: transmission electron microscopy
SYN: synaptophysin
GAP-43: growth-associated protein-43.
