# Supplementary material for: Synaptic Plasticity After Focal Cerebral Ischemia Was Attenuated by Gap26 but Enhanced by GAP-134
Source: Front Neurol. 2020 Aug 26;11:888. doi: 10.3389/fneur.2020.00888 (PMC7479336; doi:10.3389/fneur.2020.00888)
Supplement: Supplementary file 1 [file Data_Sheet_1.PDF]

Supplementary Fig. 1 Effects of Gap26 and GAP-134 on infarct area at 7 d after MCAO. Data were presented as mean ± SEM, n=6. (A) Representative TTC stained brain sections and (B) Quantitative analysis of infarct area showed that the percent infarction area significantly increased in the MCAO. However, compared with MCAO, there was no significant difference in Gap26/GAP-134 group (*$P < 0.05$ vs. sham).

Supplementary Fig. 2 Effects of Gap 26 and GAP-134 on the expression of GFAP at 7 d after MCAO. Data were shown as mean ± SEM, n=6. (A) Representative pictures of GFAP in hippocampal CA1, CA3 and DG. Blue was staining of DAPI (cell nuclei), and green was staining of GFAP. Scale bar = 20 μm. (B, C, D) Quantitative analysis showed that expression of GFAP was down-regulated by Gap26 but up-regulated by GAP-134 in hippocampal CA1, CA3 and DG (*$P < 0.05$ vs. sham, #$P < 0.05$ vs. MCAO).

Supplementary Fig. 3 Effects of Gap26 and GAP-134 on Cx43 immunostaining in lesion and hippocampus (CA1, CA3 and DG) at 7 d after MCAO. Data were shown as mean ± SEM, n=6. (A) Representative pictures of Cx43. Blue was staining of DAPI (cell nuclei), and red was staining of Cx43. Scale bar = 20 μm. (B) Quantitative analysis showed that expression of Cx43 was increased particularly around the hippocampal CA1, CA3 and DG (*$P < 0.05$ vs. cortex).

Supplementary Fig. 4 Effects of Gap26 and GAP-134 on gap junctional ultrastructure in Hippocampus. The ultrastructure of hippocampal gap junctions were analyzed using TEM at 7 d after focal ischemia. The gap junctions in sham group remained tight contacts with no visible cleft (A). MCAO impaired intercellular contacts by widening the gap between two neighboring astrocytes (B). Administration with Gap26 further deteriorated the destruction of gap junctions (C), while GAP-134 treatment attenuated the destruction of gap junctions (D). The gap junctions were indicated by black arrows. Scale bar = 500 nm.
