# Supplementary figures and images for: Synaptic Plasticity After Focal Cerebral Ischemia Was Attenuated by Gap26 but Enhanced by GAP-134
Source: Front Neurol. 2020 Aug 26;11:888. doi: 10.3389/fneur.2020.00888 (PMC7479336; doi:10.3389/fneur.2020.00888)

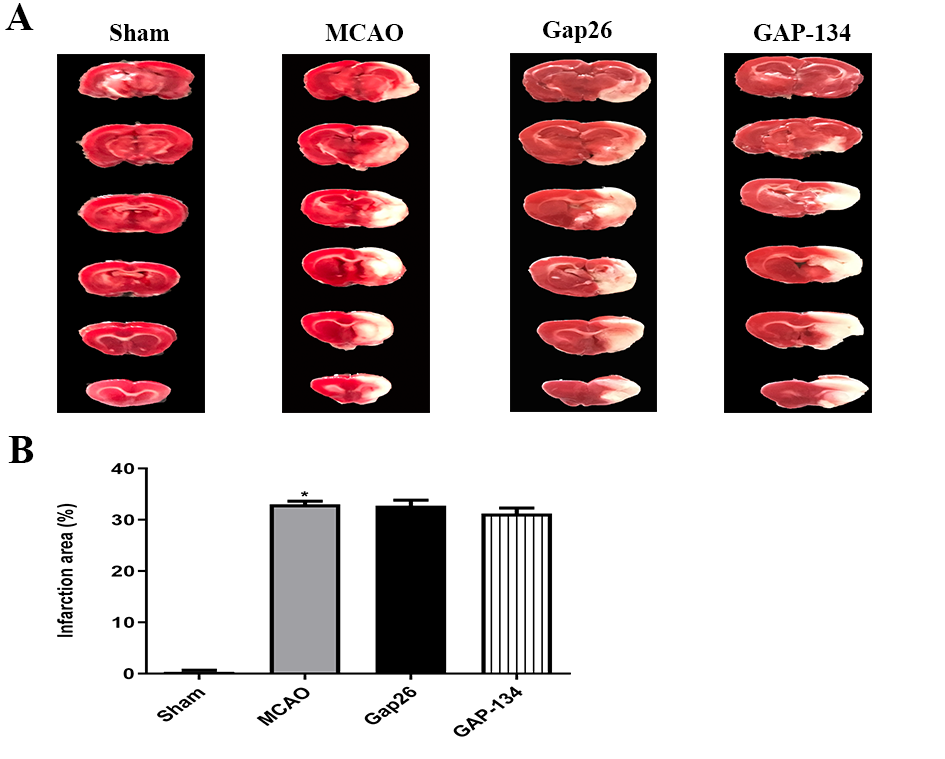

Supplement: Supplementary file 2 [file Image_1.TIF]

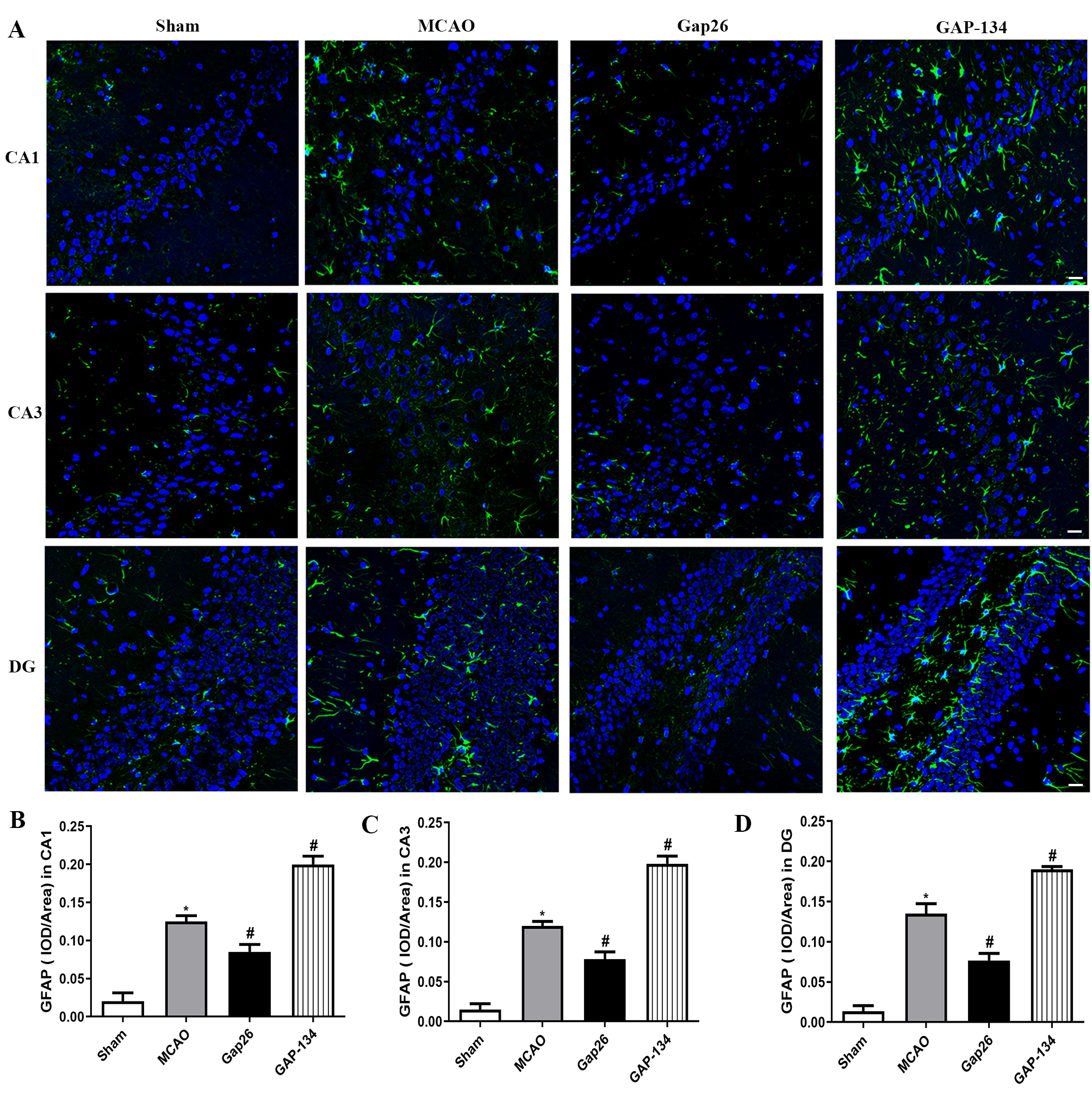

Supplement: Supplementary file 3 [file Image_2.TIF]

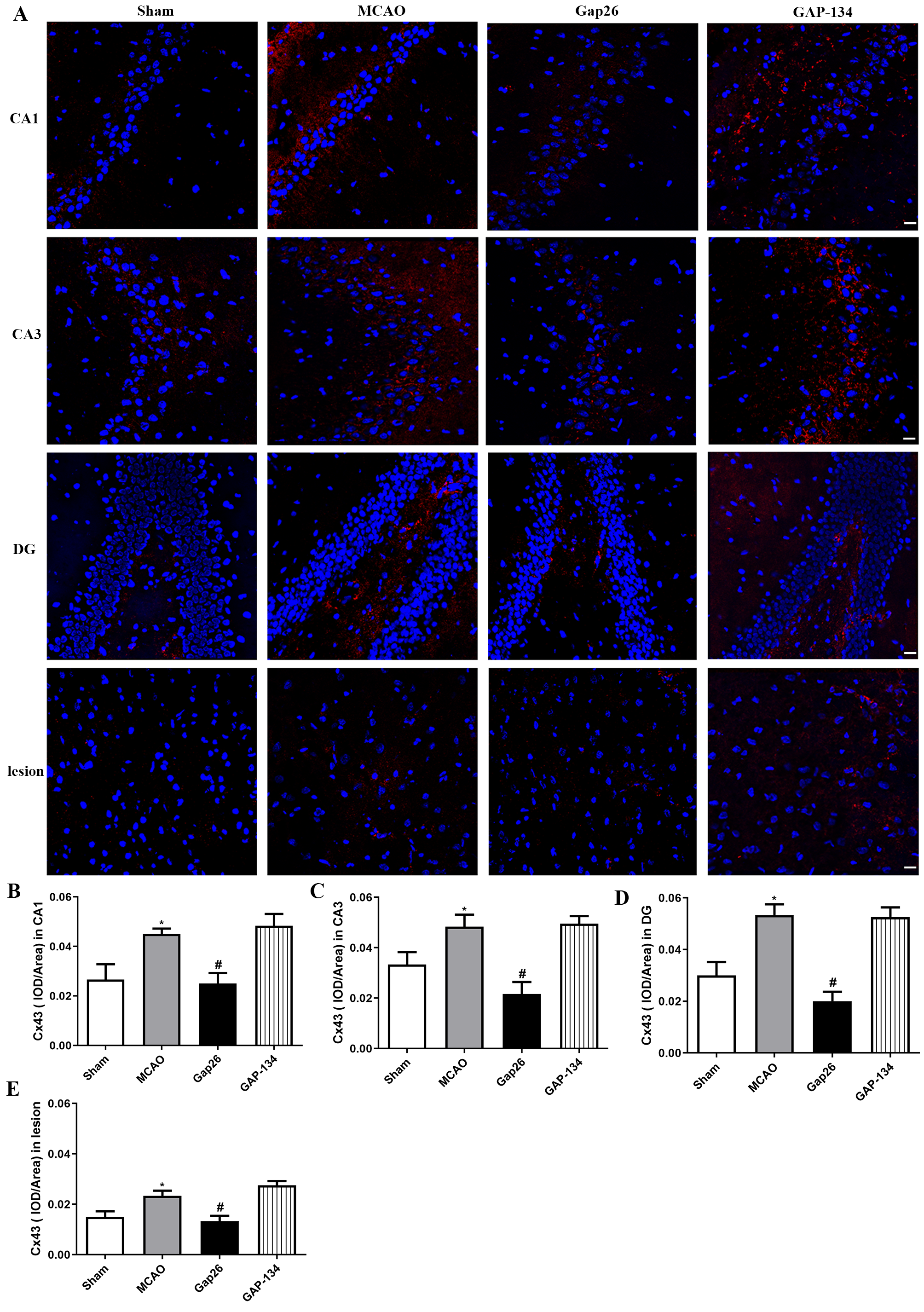

Supplement: Supplementary file 4 [file Image_3.TIF]

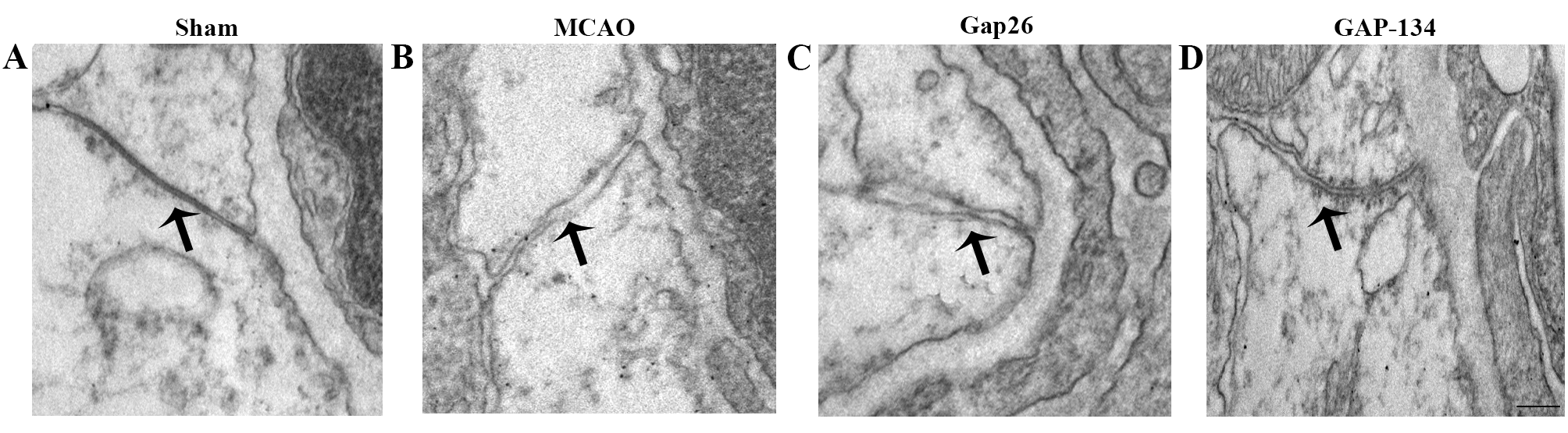

Supplement: Supplementary file 5 [file Image_4.TIF]
